# Multidisciplinary and Evidence-based Method for Prioritizing Diseases of Food-producing Animals and Zoonoses

**DOI:** 10.3201/eid1804.111151

**Published:** 2012-04

**Authors:** Marie-France Humblet, Sébastien Vandeputte, Adelin Albert, Christiane Gosset, Nathalie Kirschvink, Eric Haubruge, Fabienne Fecher-Bourgeois, Paul-Pierre Pastoret, Claude Saegerman

**Affiliations:** University of Liege, Liege, Belgium (M.-F. Humblet, S. Vandeputte, A. Albert, C. Gosset, F. Fecher-Bourgeois, C. Saegerman);; University of Namur, Namur, Belgium (N. Kirschvink);; University of Liege, Gembloux, Belgium (E. Haubruge);; Fontin, Belgium (P.-P. Pastoret)

**Keywords:** animal diseases, zoonoses, prioritization, multianalysis criteria, multidisciplinary method, evidence-based method, experts’ opinions, food-producing animals, Europe

## Abstract

To prioritize 100 animal diseases and zoonoses in Europe, we used a multicriteria decision-making procedure based on opinions of experts and evidence-based data. Forty international experts performed intracategory and intercategory weighting of 57 prioritization criteria. Two methods (deterministic with mean of each weight and probabilistic with distribution functions of weights by using Monte Carlo simulation) were used to calculate a score for each disease. Consecutive ranking was established. Few differences were observed between each method. Compared with previous prioritization methods, our procedure is evidence based, includes a range of fields and criteria while considering uncertainty, and will be useful for analyzing diseases that affect public health.

Agents that cause zoonotic diseases are infectious (transmissible) agents that not only are confined to 1 animal host but that also cause an infection (infestation) with or without clinical disease in several hosts, including humans (*1*). Nevertheless, all diseases affecting animals and humans are not strictly zoonotic but are qualified as common. Animals and humans generally contract infections from the same sources (soil, water, invertebrates, and plants). However, animals do not play an essential role in the life cycle of the etiologic agent but may contribute in various degrees to distribution and transmission of infections (*2*). According to the World Organisation for Animal Health (OIE), 75% of the emerging diseases originate in domestic or wild animals, which prompts close collaboration between animal health and public health authorities (www.oie.int/eng/edito/en_avr09.htm).

To achieve such a goal, the One Health strategy was recently developed to expand interdisciplinary collaborations and communications in all aspects of health care for humans, animals and the environment (www.onehealthinitiative.com/mission.php). Such collaborations are particularly evident when considering zoonoses. In the One Health concept, a new strategy for animal health was recently adopted by the European Union (*3*). Categorization of threats caused by animals is 1 of the pillars of this strategy. Such method aims to provide a tool for decisions in animal health issues for selecting disease-related threats that are worth being addressed by public policies, which is necessary if considering emerging infectious diseases. The method of disease prioritization has been defined as the “organization of listed diseases into a hierarchy, considering their respective impacts” (*4*).

The main objectives of a prioritization method are to optimize financial and human resources for the surveillance, prevention, control, and eradication of infectious diseases and to target surveillance for early detection of any emerging disease. Our method is based on widely used multicriteria analysis, which consists of listing criteria to assess pathogens, evaluating pathogens on the basis of these criteria (scores), determining the relative role (weight) of each criterion, and aggregating scores and weights of criteria into 1 global score per pathogen (*5*). The originality of the prioritization procedure is a reasoning based on opinions of multidisciplinary experts (weighting method) and on evidence-based data of targeted diseases (animal diseases, zoonoses, and transmissible diseases common to animals and humans).

## Methods

### Diseases

One hundred diseases were included in the prioritization exercise. Species targeted were food-producing animals: cattle, small ruminants, swine, horses, poultry (including water birds such as ducks and geese), lagomorphs, and wildlife (species common in western Europe). There were 86 diseases affecting the animal species under study and reportable to the OIE (www.oie.int/en/animal-health-in-the-world/oie-listed-diseases-2011/).

Influenza caused by highly pathogenic and low pathogenicity avian influenza viruses was considered separately. Twelve additional infectious diseases reported in Europe during September 2009 and September 2010 and reported to the International Society for Infectious Diseases (www.promedmail.org) (archives 20100215.0530, 20100803.2615, 20101227.4564, 20100729.2546, 20100330.0996, 20100111.0128, 20090912.3211, 20100620.2072, 20091217.4273, 20091004.3453, 20101106.4026, and 20100209.0442) were also included in the prioritization method: besnoitiosis, botulism, bluetongue (bluetongue virus serotype16), hantavirosis (*Puumala virus*), hepatitis E, influenza (H1N1), norovirus disease, European tick-borne encephalitis, Usutu virus disease, porcine post-weaning multisystemic wasting disease (circovirus), equine atypical myopathy, and disease caused by *Escherichia coli* O157:H7. Parafilariasis (*Parafilaria bovicola*) was also included because it is considered to be emerging in western Europe (*6*). Salmonellosis caused by *Salmonella enterica* serovar Enteritidis was considered because of its effect on public health and it is the most common serovar in the European Union (*7*). Foot-and-mouth disease (FMD) was included in the zoonotic/common category despite its low effect on public health and the limited number of human cases reported to date (*8*).

### Prioritization Criteria

Five aspects of a pathogen were considered: epidemiology, prevention/control, effects on economy/trade, zoonotic characteristics, and effect on society. The prioritization criteria were established according to a review of previous priority settings (i.e., English Department for Environment, Food and Rural Affairs [DEFRA]) (http://archive.defra.gov.uk/foodfarm/farmanimal/diseases/vetsurveillance/strategy/programme/prioritisation.htm) (*9**–**13*) and principles of evidence-based medicine, which promotes collection, interpretation, and integration of valid, essential, and applicable scientific evidence (*14*). A total of 57 criteria were retained for the prioritization method and further submitted for opinions of experts. The distribution of criteria among 5 categories was 17 for epidemiology (EP), 8 for prevention/control (PC), 16 for economy/trade, 12 for public health (PH), and 4 for society (SO). The 57 criteria are summarized in Table 1. Strict definitions were given for each coefficient and criterion and are summarized in Table 2.

**Table 1:** Fifty-seven criteria used for prioritization method for diseases of food-producing animals and zoonoses and corresponding weight attribution by experts, Europe

Category, criteria	Minimum	Average	Maximum	Fitting distribution*
Epidemiology				
Illness rate, %	2.05	7.59	18.00	Pert (2,05; 5; 18)
Case-fatality rate, %	4.19	9.13	18.00	Pert (4,19; 5; 18)
Specificity of pathogen	0.00	4.55	10.23	Uniform (0; 10,23)
Mode of transmission	0.00	8.08	23.52	Pert (0; 10; 23,52)
Incubation period	0.00	3.43	6.00	Pert (0; 2; 6)
Clinical course	0.00	2.93	6.00	Pert (0; 2; 6)
Persistence in environment	0.00	6.37	12.56	Pert (0; 5; 12,56)
Epizootic potential	0.00	9.92	22.50	Pert (0; 10; 22,5)
Evolutive characteristics of pathogen	1.89	6.39	18.00	Pert (0; 5; 18)
Clinical disease in cattle	0.00	4.52	10.71	Triang (0; 0; 10,71)
Clinical disease in small ruminants	0.00	2.37	4.74	Triang (0; 0; 4,74)
Clinical disease in swine	0.00	3.60	9.00	Triang (0; 0; 9)
Clinical disease in equines	0.00	2.99	10.00	Pert (0; 2; 10)
Clinical disease in poultry	0.00	3.42	9.00	Triang (0; 0; 9)
Clinical disease in lagomorphs	0.00	3.42	9.00	Triang (0; 0; 9)
Clinical disease in wildlife	0.00	4.39	11.25	Pert (0; 2; 11;25)
Presence/absence of vector(s)/reservoir(s) in European Union	3.07	6.90	11.25	Pert (3,07; 5; 11,25)
Intercategory weight	10.00	19.67	25.00	Pert (10; 20; 25)
Prevention/control				
Control of reservoir(s) or vector(s)	0.00	6.13	10.00	Uniform (0; 10)
Vaccination	5.00	8.63	15.00	Uniform (5; 15)
Treatment	3.00	6.63	10.00	Uniform (3; 10)
Availability and quality of diagnostic tools	5.00	7.69	10.00	Uniform (5; 10)
Knowledge of pathogenic agent	0.00	7.38	15.00	Pert (0; 5; 15)
Effectiveness of control measures other than treatment, vaccination, and control of vectors	1.00	7.26	10.00	Uniform (1; 10)
Effectiveness of prevention measures other than vaccination	5.00	8.72	12.00	Pert (5; 10; 12)
Surveillance of pathogenic agent in European Union or worldwide	4.00	7.57	15.00	Triang (4; 4; 15)
Intercategory weight	10.00	18.83	25.00	Pert (10; 20; 25)
Economy/trade				
Losses of productivity (milk, eggs, growth)	0.00	6.35	17.14	Pert (0; 9; 17,14)
Additional costs: mandatory slaughtering	0.00	5.11	12.95	Uniform (0; 12,95)
Additional costs: treatment, disinfection, labor	0.00	4.40	8.57	Pert (0; 5; 8,57)
Limited importation–exportation	0.00	4.40	8.57	Pert (0; 5; 8,57)
Disturbance of supply and demand (decrease in prices)	0.00	5.54	9.23	Uniform (0; 9,23)
Impact on adjacent sectors (tourism)	0.00	4.80	17.14	Triang (0; 0; 17,14)
Impact on cattle industry	0.00	3.29	17.14	Triang (0; 0; 17,14)
Impact on small ruminants industry	0.00	3.09	8.57	Uniform (0; 8,57)
Impact on swine industry	0.00	1.87	8.57	Triang (0; 0; 8,57)
Impact on equine industry	0.00	2.81	8.57	Triang (0; 0; 8,57)
Impact on poultry industry	0.00	1.77	8.57	Triang (0; 0; 8,57)
Impact on rabbit industry	0.00	2.81	8.57	Triang (0; 0; 8,57)
Impact on wildlife industry	0.00	2.81	8.57	Triang (0; 0; 8,57)
Zoonotic impact (cost of illness)	0.00	2.17	8.96	Triang (0; 0; 8,57)
Zoonotic impact (costs of prevention per person)	0.00	4.59	10.75	Triang (0; 0; 10,75)
Intercategory weight	10.00	23.00	30.00	Uniform (10; 30)
Public health				
Zoonotic/common agent	0.00	7.81	20.00	Pert (0; 10; 20)
Classification of zoonoses	0.00	5.51	11.25	Pert (0; 6; 11,25)
Disease knowledge in humans	2.40	7.27	11.25	Pert (2,4; 5; 11,25)
Illness rate, %	1.01	8.08	12.00	Uniform (1,01; 12)
Case-fatality rate, %	1.01	9.46	18.00	Pert (1,01; 10; 18)
Mode of transmission	0.00	5.71	10.59	Pert (0; 5; 10,59)
After effects or negative impact on the patient quality of life	5.29	8.88	12.00	Uniform (5,29; 12)
Presence of a control plan	3.03	5.37	6.99	Pert (3,03; 5; 6,99)
Epidemic potential	5.29	8.98	12.13	Uniform (5,29; 12,13)
Vaccination	5.00	7.49	11.25	Uniform (5; 11,25)
Treatment	5.00	7.35	11.25	Uniform (5; 11,25)
Availability and quality of diagnostic tools	5.00	8.10	15.17	Triang (5; 5; 15,17)
Intercategory weight	20.00	24.67	30.00	Uniform (20; 30)
Society				
Lower human consumption of animals	0.00	7.19	15.00	Uniform (0; 15)
Perception of problem by consumer	0.00	6.92	12.00	Uniform (0; 12)
Potential impact on media	0.00	6.50	20.00	Uniform (0; 20)
Impact on animal welfare and biodiversity	1.00	9.38	20.00	Uniform (0; 20)
Intercategory weight	8.00	13.83	20.00	Uniform (8; 20)

*Pert, program evaluation and review technique; uniform, uniform probability distribution; triang, triangular distribution (www.palisade.com/downloads/manuals/EN/RISK5_EN.pdf).

**Table 2:** Fifty-seven criteria used for ranking diseases of food-producing animals and zoonoses classified by category, Europe*

Epidemiology		Score																					
Ranking	Criteria	0	1				2				3			4			5					6	7
1	Illness rate, %		<1				1–10				11–30			31–50			51–70					71–90	>90
2	Case-fatality rate, %		<1				1–10				11–30			31–50			51–70					71–90	>90
3	Agent specificity		1 host species				2 host species				3 host species			4 host species			>4 host species						
4	Mode of transmission		No vector-borne transmission (not contagious)				Contamination by direct contact				Contamination by indirect contact			Vector-borne transmission			Airborne contamination						
5	Incubation period	Not applicable: clinical disease never reported in species considered in the study	<1 d				1–7 d				8–14 d			15–30 d			1–6 mo					>6–12 mo	>12 mo
6	Clinical course	Not applicable: clinical disease never reported in species considered in the study	<1 d				1–7 d				8–14 d			15–30 d			1–6 mo					>6–12 mo	>12 mo
7	Environmental persistence	None: no persistence in the environment, no vector(s) or wildlife reservoir(s) identified	Rare: anecdotal isolation in a potential vector(s) or the environment				No data available on presence/survival of pathogenic agent in reservoir(s), vector(s) or the environment				Wildlife reservoir(s)/vector(s): pathogen agent persistent in wildlife reservoir(s) and/or vector(s)			Environment: agent naturally surviving in the environment (soil, water)									
8	Epizootic potential		Never: only sporadic cases, epizootics never reported				Rare: most cases are sporadic; possibility of localized epizootic if conditions are ideal: e.g., abnormal multiplication of reservoir(s) and/or vector(s)				Localized: pathogen characterized by localized epizootic potential essentially related to the transmission mode: e.g., food-borne diseases			(Inter)national: epizootic characteristics well known after introduction, possibility of wide spatiotemporal expansion									
9	Evolutive characteristics of pathogen		Null: stability of pathogen, stable pathogen–vector(s)/pathogen–reservoir(s) relationships (no impact on pathogenicity)				Rare: some mutations/reassortments observed but without any impact on pathogenicity, stable pathogen–vector(s)/pathogen–reservoir(s) relationships				Moderate/not determined: pathogen not characterized for evolutive characteristics yet (recently discovered, limited means of study), mutations with limited consequences on its virulence; stable pathogen–vector(s)/pathogen–-reservoir(s) relationships			Frequent: genetic variability during replication cycles more or less defined; variability of pathogenicity, species affected, reservoir(s), and vector(s)			High: pathogen has a high mutation rate/frequent genetic reassortments and creation of new pathogenic variants at each cycle: variable pathogenicity, host(s), reservoir(s), and vector(s)						
10	Cattle	Pathogen never reported as etiologic agent of clinical disease in that species	Accidental: few clinical cases reported, only if conditions are favorable (wound, traumatism, favorable environmental conditions)				Rare: clinical disease reported in few cases and no need for favorable conditions				Occasional: clinical disease occasionally reported and no need for favorable conditions			Frequent: clinical disease frequently reported in that species, but not specifically (multispecies pathogen)			Specific: clinical disease only reported in that species					Reservoir species	
11	Small ruminants	Pathogen never reported as etiologic agent of clinical disease in that species	Accidental: few clinical cases reported, only if conditions are favorable (wound, traumatism, favorable environmental conditions)				Rare: clinical disease reported in few cases and no need for favorable conditions				Occasional: clinical disease occasionally reported and no need for favorable conditions			Frequent: clinical disease frequently reported in that species, but not specifically (multispecies pathogen)			Specific: clinical disease only reported in that species					Reservoir species	
12	Swine	Pathogen never reported as etiologic agent of clinical disease in that species	Accidental: few clinical cases reported, only if conditions are favorable (wound, favorable environmental conditions)				Rare: clinical disease reported in few cases and no need for favorable conditions				Occasional: clinical disease occasionally reported and no need for favorable conditions			Frequent: clinical disease frequently reported in that species, but not specifically (multispecies pathogen)			Specific: clinical disease only reported in that species					Reservoir species	
13	Equine	Pathogen never reported as etiologic agent of clinical disease in that species	Accidental: few clinical cases reported, only if conditions are favorable (wound, traumatism, favorable environmental conditions)				Rare: clinical disease reported in few cases and no need for favorable conditions				Occasional: clinical disease occasionally reported and no need for favorable conditions			Frequent: clinical disease frequently reported in that species, but not specifically (multispecies pathogen)			Specific: clinical disease only reported in that species					Reservoir species	
14	Poultry	Pathogen never reported as etiologic agent of clinical disease in that species	Accidental: few clinical cases reported, only if conditions are favorable (wound, traumatism, favorable environmental conditions)				Rare: clinical disease reported in few cases and no need for favorable conditions				Occasional: clinical disease occasionally reported and no need for favorable conditions			Frequent: clinical disease frequently reported in that species, but not specifically (multispecies pathogen)			Specific: clinical disease only reported in that species					Reservoir species	
15	Lagomorphs	Pathogen never reported as etiologic agent of clinical disease in that species	Accidental: few clinical cases reported, only if conditions are favorable (wound, traumatism, favorable environmental conditions)				Rare: clinical disease reported in few cases and no need for favorable conditions				Occasional: clinical disease occasionally reported and no need for favorable conditions			Frequent: clinical disease frequently reported in that species, but not specifically (multispecies pathogen)			Specific: clinical disease only reported in that species					Reservoir species	
16	Wildlife	Pathogen never reported as etiologic agent of clinical disease in that species	Accidental: few clinical cases reported, only if conditions are favorable (wound, traumatism, favorable environmental conditions)				Rare: clinical disease reported in few cases and no need for favorable conditions				Occasional: clinical disease occasionally reported and no need for favorable conditions			Frequent: clinical disease frequently reported in that species, but not specifically (multispecies pathogen)			Specific: clinical disease only reported in that species					Reservoir species	
17	Presence/absence of vector(s) and/or reservoir(s) in EU	Not vector-borne disease and/or no known reservoir	Absence of vector(s)/reservoir(s) in EU				Localized presence: reservoir(s) and/or vector(s) in a limited area of >1 member states				Mediterranean region/northern Europe/central Europe: vector(s) and/or reservoir(s) in 1 of these 3 regions, each one covering several member states, presence linked to bioclimatic preferences			Mediterranean region, northern Europe/northern Europe, central Europe: vector(s) and/or reservoir(s) in 1 of both regions according to bioclimatic preferences			Generalized repartition: repartition of vector(s) and/or reservoir(s) in the entire EU (few bioclimatic specificities)						
Prevention–control		Score																					
Ranking	Criteria	0	1					2					3							4			
1	Control of reservoir(s) and/or vector(s)	Not applicable: no vector-borne transmission and/or no reservoir(s) known to date	Effective: limited reservoir(s), easy to identify; effective control measures and trapping; reservoir(s)/vector(s) with limited demographic and geographic repartition; extensive scientific knowledge of vector(s)/reservoir(s); possibility of integrated control method					Limited: limited reservoir(s), easy to identify; effective control measures and trapping but not applicable at a large scale; reservoir(s)/vector(s) with a limited demographic and geographic repartition; extensive scientific knowledge of vector(s)/reservoir(s); no integrated control method					Possible but poorly/not effective: reservoirs easy to identify but numerous; control measures and trapping poorly effective (poorly active molecule(s); resistances and/or negative impact on environment); reservoir(s)/vector(s) with a limited demographic and geographic repartition; no scientific knowledge of vector(s)/reservoir(s); no integrated control method							Absent/impossible: vector(s)/reservoir(s) not identified; no effective control measure against vector(s) (no active molecule, ineffective trapping); strong demography and/or wide repartition of vector(s) and/or reservoir(s); no scientific knowledge of vector(s)/reservoir(s); no integrated control method			
2	Vaccination	Not applicable: clinical disease never reported in species considered in the study	Commercialized: commercial vaccine available on a global scale					Local/monospecies: vaccine available at a regional/national scale and/or for a targeted species (not systematically available for a global control plan)					Experimental: experimental vaccine, not commercialized; severe adverse reaction when applied; limited protector effect							Absence: no vaccine available for use in species considered in the study, no experimental vaccine			
3	Treatment	Not applicable: clinical disease never reported in species considered in the study	Available/effective: effective treatment available; recommended in cases of infection; economical and rational from a zootechnical point of view					Available but not recommended: masks clinical course of disease; contrary to the control plan; not justified economically or from a zootechnical point of view					Available but poorly/not effective: treatment with a limited effectiveness; severe adverse reactions; experimental or empirical treatment							Absence: no effective treatment available, no experimental treatment available			
4	Availability and quality of diagnostic tools		High: field test(s) available and easy to use, and highly discriminating sensitivity and specificity					Moderate: tests only used in local/regional laboratories					Low: tests only used in specialized laboratories/national reference laboratory							Absence: no diagnostic tools available			
5	Knowledge of pathogen		Very high: extensive scientific knowledge of pathogen, extensive scientific literature available on its biology: transmission mode, knowledge of vector(s), infectivity					High: detailed scientific knowledge of pathogen but conflicting scientific results; some elements of pathogen biology are still not elucidated					Moderate: limited scientific knowledge of pathogen because it is still being characterized; pathogen recently discovered/isolated but belonging to a well known and studied family of pathogens; pathogen characterized by multiple variants not characterized							Low: no scientific knowledge of pathogen (multiplication, infectivity, incubation period, transmission mode); pathogen recently discovered or emerging			
6	Effectiveness of control measures other than treatment, vaccination, and vector(s)/ reservoir(s) control		High: effectiveness of implemented control measures (quarantine, slaughter, and restriction area); effective epidemiologic investigation (origin of the infection rapidly identified and quick implementation of control measures)					Moderate: effectiveness of implemented control measures (quarantine, slaughter, and restriction area); epidemiologic investigation poorly conclusive (incomplete traceability of animals and by-products)					Low: limitation of control measures implemented (quarantine, slaughter, and restriction area), limiting dissemination of pathogen; epidemiologic investigation inconclusive							Null: ineffectiveness of implemented control measures (quarantine, slaughter, and restriction area) and/or control measures not indicated because of characteristics of pathogen; epidemiologic investigation inconclusive			
7	Effectiveness of prevention other than vaccination and control of vector(s)/ reservoir(s)		High: sanitary certificate; effective traceability of animals and by-products; effective disinfection measures; no contact between domestic and wild animals; effective biosecurity measures					Moderate: no sanitary certificate; effective traceability of animals and by-products; effective disinfection measures; limited or incomplete possibilities to restrict contacts between domestic and wild animals; effective biosecurity measures					Low: no sanitary certificate; incomplete traceability of animals and by-products; ineffective disinfection measures; incomplete restriction of contacts between domestic and wild animals; ineffective biosecurity measures							Null: no sanitary certificate; no traceability of animals and by-products; ineffective disinfection measures; no restriction of contact between domestic and wild animals; ineffective biosecurity measures			
8	Surveillance of pathogen		Generalized: surveillance implemented by all EU member states (even worldwide surveillance)					Member states at risk: surveillance of pathogen in >1 neighboring member states and in those where epizootics were recently reported					Outside EU: pathogen surveyed in non-EU regions							Absent: no surveillance of pathogen			
Economy/trade		Score																					
Ranking	Criteria	0		1								2						3					
Individual data (herd/farmer)																							
1	Losses of productivity (milk, eggs, growth)	Null: no impact on animal productivity		Low: losses of productivity <20%								Moderate: losses of productivity of 20%–50%						Severe: losses of productivity >50%					
2	Additional costs: mandatory slaughtering	Not required		Outbreaks only								Outbreaks and restriction areas											
	Additional costs: treatment, disinfection			Low: treatment not required (e.g., slaughtering justified from an economic point of view) or absent (virus), application of basic sanitary measures (disinfection, footbath)								Moderate: spontaneous resolution of cases, only the animals with serious clinical signs require treatment, application of basic sanitary measures (disinfection, footbath)						High: systematic treatment of animals with clinical signs; application of stricter sanitary measures					
3	Additional costs: vaccination			Low: no vaccination advocated or no vaccination available								Moderate: vaccination not mandatory but possible in particular cases, e.g., avian sector						High: mandatory vaccination					
Global (sector/market)																							
4	Limitation of importation–exportation	Absent: no impact on the importation/exportation of animal and/or by-products		Local: restrictions of animal and/or by-products movements limited to surveillance areas implemented when an outbreak is confirmed								Regional: animal and/or by-products movements limited in an area greater than the surveillance zone but only in 1 member state						International: perturbation/limitation of importations/exportations of animal and by-products between several member states and/or between member states and countries outside the EU					
5	Disturbance of supply and demand (decrease in prices)	Absent: no impact on supply and demand		Low: temporary disturbance of supply and demand in a limited area and low impact on prices								Moderate: temporary disturbance of supply and demand and decrease in prices <30% in >1 member states						High: major disturbance of supply and demand and decrease in prices >30% affecting several member states					
6	Impact on related sectors (tourism, animal feeds)	Absent: no impact on related sectors		Low: turnover reduction <20% in >1 related sectors								Moderate: turnover reduction 20%–50% in >1 related sectors						High: turnover reduction >50% in >1 related sectors					
7	Impact on cattle industry	Absent: no impact on cattle industry		Low: increased spends and/or decreased benefits <20% compared with situation before beginning of epizootics								Moderate: increased spends and/or decreased benefits between 20% and 50% compared with situation before beginning of epizootics						High: increased spends and/or decreased benefits >50% compared with situation before beginning of epizootics					
7	Impact on small ruminants industry	Absent: no impact on small ruminants industry		Low: increased spends and/or decreased benefits <20% compared with situation before beginning of epizootics								Moderate: increased spends and/or decreased benefits between 20% and 50% compared with situation before beginning of epizootics						High: increased spends and/or decreased benefits >50% compared with situation before beginning of epizootics					
7	Impact on swine industry	Absent: no impact on swine industry		Low: increased spends and/or decreased benefits <20% compared with situation before beginning of epizootics								Moderate: increased spends and/or decreased benefits between 20% and 50% compared with situation before beginning of epizootics						High: increased spends and/or decreased benefits >50% compared with situation before beginning of epizootics					
7	Impact on equine industry	Absent: no impact on equine industry		Low: increased spends and/or decreased benefits <20% compared with situation before beginning of epizootics								Moderate: increased spends and/or decreased benefits between 20% and 50% compared with situation before beginning of epizootics						High: increased spends and/or decreased benefits >50% compared with situation before beginning of epizootics					
7	Impact on poultry industry	Absent: no impact on poultry industry		Low: increased spends and/or decreased benefits <20% compared with situation before beginning of epizootics								Moderate: increased spends and/or decreased benefits between 20% and 50% compared with situation before beginning of epizootics						High: increased spends and/or decreased benefits >50% compared with situation before beginning of epizootics					
	Impact on lagomorph industry	Absent: no impact on poultry industry		Low: increased spends and/or decreased benefits <20% compared with situation before beginning of epizootics								Moderate: increased spends and/or decreased benefits between 20% and 50% compared with situation before beginning of epizootics						High: increased spends and/or decreased benefits >50% compared with situation before beginning of epizootics					
7	Impact on wildlife industry	Absent: no impact on wildlife industry		Low: increased spends and/or decreased benefits <20% compared with situation before beginning of epizootics								Moderate: increased spends and/or decreased benefits between 20% and 50% compared with situation before beginning of epizootics						High: increased spends and/or decreased benefits >50% compared with situation before beginning of epizootics					
Cost of disease in humans																							
8	Zoonotic impact (cost of illness)	Absent: nonzoonotic or common† disease		Low: medical consultation facultative, hospitalization not required, treatment for most severe clinical cases with conventional drugs, maximum incapacity 7 d								Moderate: medical consultation necessary, hospitalization of most severe clinical cases, systematic and adapted treatment with conventional drugs, incapacity 8–4 d						High: medical consultation necessary, systematic hospitalization but of variable duration, required and adapted treatment with second line drugs, incapacity >14 d, quarantine may be required					
9	Zoonotic impact (costs of prevention per person)	Absent: nonzoonotic or common disease		Low: vaccination not advocated, simple and low-cost preventive measures (handwashing, mask carrying, insect repellents)								Moderate: vaccination of populations at risk (YOPI), simple and low-cost preventive measures (handwashing, mask carrying, insect repellents)						High: generalized vaccination recommended, restricting and expensive preventive measures (thermograph, quarantine, home containment)					
Public health		Score																					
Ranking	Criteria	0	1			2				3						4			5			6	7
1	Zoonotic/ common agent†	Not zoonotic or common	Accidental: human clinical disease only when favorable conditions are set (YOPI, high infection pressure, practices at risk, unusual transmission route)			Rare: human clinical disease reported in a minority of cases, without necessity of favorable conditions				Frequent: clinical disease often reported in man (multi-species pathogen) without need for favorable conditions						Systematic: clinical disease systematically reported in humans							
2	Classification of zoonoses	Not zoonotic or common	1: transmission from wild animals to humans			1+: transmission from wild animals to humans with further human-to-human transmission(s)				2: transmission from wild animals to domestic animals to humans						2+: transmission from wild animals to domestic animals to humans, and further human-to-human transmission(s)							
3	Disease knowledge in humans	Not zoonotic or common	Very high: deep scientific knowledge of pathogen, extensive scientific literature available on its biology: transmission mode, knowledge on vector(s), infectivity			High: detailed scientific knowledge of pathogen but conflicting scientific results; some elements of pathogen biology are still not elucidated				Moderate: limited scientific knowledge of pathogen because it is still being characterized; pathogen recently discovered/isolated but belonging to a well-known and studied family of pathogens; pathogen has multiple variants not characterized						Low: no scientific knowledge of pathogen (multiplication, infectivity, incubation period, transmission mode); pathogen agent recently discovered or emerging							
4	Illness rate, %	Not zoonotic or common	<1			1–10				11–30						31–50			51–70			71–90	>90
5	Case-fatality rate, %	Not zoonotic or common	<1			1–10				11–30						31–50			51–70			71–90	>90
6	Mode of contamination	Not zoonotic or common	No vector-borne transmission (not contagious)			Contamination by direct contact				Contamination by indirect contact						Vector-borne transmission			Airborne contamination				
7	Aftereffects or negative impact on the patients' quality of life	Not zoonotic or common	Null: no after effects			Moderate: % disability <30% but no loss of autonomy				Severe: after effects not enabling a professional activity but no loss of autonomy						Very severe: unable to perform professional activities, loss of autonomy, and personal assistance necessary							
8	Control plan (vaccination, determination of populations at risk, surveillance of the disease, definition of areas at risk)	Not zoonotic or common	Worldwide (EU and other countries): international and coordinated control plan (member states and third-world countries)			Generalized (EU): coordinated control plan implemented in all member states				Targeted: coordinated control plan implemented in >1 member state(s) at risk						Extracommunautary: absence of a control plan in EU but implemented in third-world countries			Absent: no control plan elaborated and implemented				
9	Epidemic potential	Not zoonotic or common	Never: only sporadic cases, epidemics never reported			Rare: most cases are sporadic but when favorable conditions are set, possibility of localized epidemics, e.g., abnormal multiplication of reservoir(s) and/or vector(s)				Localized: pathogen characterized by localized epidemic; potential pathogenicity essentially related to transmission mode (e.g., food-borne diseases)						(Inter)national: epidemic characteristics well known after introduction, possible a wide spatiotemporal expansion							
10	Vaccination	Not zoonotic or common	Commercialized: commercial vaccine available on a global scale			Local/monospecies: vaccine available at a regional/national scale (not systematically available for a global control plan)				Experimental: experimental vaccine, not commercially available; severe adverse reaction when applied; limited protector effect						Absence: no commercially available or experimental vaccine							
11	Treatment	Not zoonotic or common	Existing/effective: effective treatment commercially available			Available but not recommended: major side effects				Available but poorly effective: treatment with limited effectiveness, partial resistance of pathogen or experimental treatment						Absent: no commercially available or experimental treatment							
12	Availability and quality of diagnostic tools	Not zoonotic or common	High: field test(s) available and easy to use with highly discriminating sensitivity and specificity			Moderate: tests only used in local/regional laboratory				Low: tests only used in specialized laboratories/national reference laboratory						Absence: no diagnostic tools available							
Society		Score																					
Ranking	Criteria	0			1				2						3						4		
1	Lower human consumption of animals	No: no impact on consumption			Low: impact on consumption and a decrease <20% compared with previous consumption				Moderate: impact on consumption and a decrease of 20%–50% compared with previous consumption						High: impact on consumption and a decrease >50% compared with previous consumption								
2	Perception of problem by the consumer (problem poorly known or unknown, problem poorly controllable or uncontrollable, affects a sensitive public)	Not zoonotic or common			Null: clear perception by the consumer; problem well known, controllable, and no impact on the family; short-term effect; does not affect a sensitive public (children, pregnant women)				Low: clear perception by the consumer; problem well known, controllable, and no impact on the family; long-term effect; does not affect a sensitive public (children, pregnant women)						Moderate: clear perception by the consumer; problem poorly known, controllable, with an impact on the family; long-term effect; affects a sensitive public (children, pregnant women)						High: bad perception by the consumer; problem poorly known, difficult to control, with an impact on the family; long-term effect; affects a sensitive public (children, pregnant women)		
3	Potential impact of media	Null: no impact of media on consuming habits			Low: short-term and minor impact on consuming habits				Moderate: long-term but minor impact on consuming habits						High: major and long-lasting impact on consuming habits (rejection of a particular by-product)								
4	Impact on animal welfare and biodiversity	Null: no impact on animal welfare and biodiversity: no slaughtering, no specific control measures applied to wildlife, no quarantine or containment of animals			Low: no slaughtering but limited control measures and limited containment of species at risk (domestic and wild animals)				Moderate: selective slaughtering of animals showing clinical signs in outbreaks, control and containment of species at risk (domestic and wild animals)						High: systematic slaughtering of domestic and wild animals (outbreaks and surveillance zones), mandatory quarantine, containment of domestic animals at risk								

*EU, European Union; YOPI, young, old, pregnant, immunosuppressed. †Common, pathogen able to cause a clinical disease in humans and animals but without a zoonotic characteristic (common source of contamination).

A particular classification of zoonoses based on interactions between host species is included as a criterion in the PH category. Type 1 diseases are those transmitted from wildlife to humans, type 1+ are transmitted from wildlife to humans with additional human-to-human transmission, type 2 are transmitted from wildlife to domestic animals and then to humans, and type 2+ are diseases transmitted from wildlife to domestic animals and then to humans, with further human-to-human transmission (*15*).

### Coefficients of Criteria

For each criterion, a coefficient of 0–7 was assigned to each option (Table 2) according to its role, effect, or rate. Coefficients were correlated with severity: the more severe the effect, the higher the coefficient. For example, a case-fatality rate <1% has a coefficient of 1 and a case-fatality rate >90% has a coefficient of 7. For nonzoonotic agents, a coefficient of 0 was fixed for criteria included in the PH category.

For each disease, evidence-based information concerning the 57 criteria was obtained from different sources, including use of OIE and Iowa State University (Ames, IA, USA) fact sheets and consultation with websites of international organizations (OIE, World Health Organization, European Centre for Diseases Prevention and Control), and Centers for Disease Control and Prevention). Web site searches for peer-reviewed literature were conducted in PubMed and the Thomson Reuters (formerly Institute for Scientific Information) Web of Knowledge. Useful information was also obtained from scientific reference books (*16**,**17*). Searches enabled collection of ≈100% of information needed for the 57 criteria for the 100 diseases.

### Multidisciplinary Panel of Experts

The main characteristic of the panel of experts consulted within the framework of the project was its multidisciplinarity. A total of 74 international experts were selected according to their field of expertise: veterinary and human epidemiologists, chief veterinary officers, economists, medical doctors, sociologists, and experts in public health and animal welfare.

Two tasks were assigned to the experts. First, they were asked to give their opinion on the pertinence of criteria proposed by indicating their degree of agreement. They were then asked to assign a score of 1 if they strongly disagreed with a criterion, 2 if they disagreed, 3 if they simply agreed, and 4 if they strongly agreed. In instances of strong disagreement, experts were asked to justify their decision and propose alternative options. Second, they were asked to weight criteria. Because all criteria do not have the same role in terms of risk and consequences within the same category, experts were thereafter asked to apply a Las Vegas method between the criteria according to their relative roles (or weights) (*18*). Because the number of criteria differs between categories, the number of points to distribute was proportional to the number of criteria per category: 90 for EP and PH, 60 for PC and EC, and 30 for SO. This method was necessary to prevent criteria classified as major by experts (in terms of points distributed) from receiving fewer points because they belonged to a category that included more criteria. Finally, 6 multicategory experts from international organizations were asked to apply the Las Vegas method for intercategory weighting by distributing 100 points between the 5 categories of criteria.

### Weighting of Scores and Ranking According to Experts

After experts weighted the different criteria, an overall weighted score was calculated for each disease (*19*). To perform the ranking, we used an aggregation method that combined 2 types of weighting. First, intracategory weighting consisted of multiplying the coefficient (0–7) allocated to the criterion by the average of points (weight) distributed by the experts for that criterion. A global score for a category was obtained by summing the weighted scores obtained for each criterion. Second, multicategory experts performed intercategory weighting. The mean number of points allocated by these experts to each category of criteria (weight) was multiplied by the global score obtained for each category after the first weighting. The overall weighted score of each disease resulted from the summation of global scores obtained from the 5 categories, as shown in the equations OWS = Σ_cat_ (GSC_j_ × IrW_j_) and GSC_j_ = Σ_crit_ (C_i_ × IaW_i_), where OWS = overall weighting score of a pathogen, GSC_j_ = global score of a category of criteria, IrW_j_ = intercategory weight for each category of criteria (average for deterministic method), C_i_ = initial coefficient per criterion, IaW_i_ = intracategory weight for each criterion (average for deterministic method), Cat = categories of criteria, and Crit = criteria.

### Uncertainty Analysis

Uncertainty of the weighting method was estimated by using a probabilistic method. All weights were converted into a function (Table 1) and computed by using @Risk software version 5.5 (Palisade Corporation, Ithaca, NY, USA). Functions were then combined through an aggregation method by using a Monte-Carlo simulation with 1,000 iterations to obtain a function of the overall weighted scores per disease with a 95% CI.

### Classification of Diseases by Using Classification and Regression Tree Analysis

Different groups of roles were identified by using classification and regression tree (CART) (www.salford-systems.com) analysis with overall weighted scores per disease as input (probabilistic method). This widely used method developed by Breiman et al. (*20*) can be applied to analyze either categorical (classification) or continuous data (regression) (*11**,**12**,**20**,**21*). In this report, regression tree models were used as the target variable and disease role was the continuous variable (*22*). The aim of these models was to obtain subgroups with minimal within-variance (grouping diseases with a similar role) by using cross-validation (*11**,**23*). Default settings of the software described by Steinberg and Golowya were used to develop the regression tree (*24*).

## Results

### Expert Opinions for Criteria

The response rate of the 74 experts on the procedure was 54% (i.e., 40 replies). Profiles of the experts are shown in Table 3. Experts were classified according to the different categories of criteria as follows: 18 for EP, 16 for PC, 14 for EC, 10 for PH, and 13 for SO. Opinions of 6 cross-category experts who assessed all categories of criteria (multicategory experts) were also included individually in each category.

**Table 3:** Characteristics of 40 experts who analyzed diseases of food-producing animals and zoonoses, Europe*

Expert	Location	Sex	Background	Country	Field of expertise	Keywords	Categories of criteria
H. Amory	Univ	F	DVM, PhD, University Professor (Faculty of Veterinary Medicine)	Belgium	Equine internal medicine	Internal medicine, cardiology, echocardiography, infectious diseases	EP
J.-M. Bouquiau	Min	M	Agronomy Engineer, University Professor (Faculty of Agronomy)	Belgium	Agriculture economy	Agricultural economist, evaluation of losses, farmer, industry, prevision of indigenous brut production	EC
S. Brunet	Univ	M	Lic Political Science and Public Administration, PhD, Instructor in Political Science	Belgium	Sociology	Risk sociology, participative methods, interactions science/society	SO
Y. Coppieters	Univ	M	MD, PhD, University Professor (School of Public Health)	Belgium	Public health	Epidemiology, health promotion, adult formations, cardiovascular diseases	PH
G. Czaplicki	Lab	M	DVM, Head of a veterinary diagnostic Laboratory	Belgium	Laboratory diagnosis	Animal serology, bovine pathology, swine pathology, epidemiology, animal infectiology	EP
X. Demarche	EuroC	M	DVM, Administrator European Institution	International†	Agriculture economy	Agriculture, animal health, food hygiene, community expenditures, international trade	EC
M. Dominguez	FAO	F	DVM, FAO Global Early Warning System, Associate Professional Officer	Italy	Animal epidemiology	Epidemiology, veterinary public health, surveillance, arboviruses, international health	EP, EC, PC, PH, SO
P.-V. Drion	Univ	M	DVM, PhD, University Professor (Experimental methods of laboratory animals and ethics in animal experiments, University of Liege)	Belgium	Animal welfare	Animal ethics, laboratory animals, animal experimentation	SO
B. Duquesne	Univ	F	Lic Agronomy, PhD, University Professor (Faculty of Agronomy)	Belgium	Agriculture economy	Veterinarian, consumption, food safety, economy, agroalimentary industry	EC
F. Fecher	Univ	F	Lic Economics, PhD, University Professor (Faculty of Economics)	Belgium	Economy	Health economy, social economy, health systems, hospital financing	EC
S. Geerts	Univ	M	DVM, PhD, Dipl, EVPC, University Professor (Institute of Tropical Medicine, Animal Health Department, head of the Unit of Veterinary Protozoology)	Belgium	Parasitology	Tropics, parasitology, zoonosis, trypanosomiasis, cysticercosis	EP, EC, PC, PH, SO
J. Godfroid	Univ	M	DVM, PhD, University Professor (Professor at the Norwegian School of Veterinary Science, Section of Arctic Veterinary Medicine; Extraordinary Professor at the University of Pretoria, Faculty of Veterinary Science, Department of Veterinary Tropical Diseases)	Norway, South Africa	Bacteriology	Brucellosis, tuberculosis, cattle, diagnosis	EP, EC, PC, PH, SO
C. Gosset	Univ	F	MD, PhD, University Professor (School of Public Health, Faculty of Medicine)	Belgium	Public health	Public health, epidemiology, health observatory, health care, economics of health	EP, PC, EC, PH
L. Hallet	CVO	M	DVM, former Chief Veterinary Officer	Belgium	Control	Reportable diseases, veterinarian, rabies vaccination	EP
A. Huberty	CVO	M	DVM, Chief Veterinary Officer	Luxemburg	Control	Biosecurity, epidemiologic, surveillance, vigilance, risk assessment, identification	EP, EC, PC, PH, SO
N. Kirschvink	Univ	F	DVM, PhD, University Professor (Department of Veterinary Medicine; Unit of Integrated Research in Veterinary Medicine, Namur Research Institute for Life Sciences)	Belgium	Small ruminants	Animal production, sheep reproduction, ovine medicine, pathophysiology, respiratory diseases	EP
A. Leblond	Univ	F	DVM, PhD, Dipl European College Equine Internal Medicine, University Professor (Department of Horse Internal Medicine); RESPE scientific committee; ANSES	France	Equine internal medicine	Internal medicine, equids, epidemiology, infectious diseases, neurology	EP, PC
M. Lefèvre	Univ	F	Lic Economics, PhD (Department of Economics)	Belgium	Agriculture economy	Development economy, microeconomy, agricultural economy, dairy cattle,western Africa	EC
L. Lengelé	CVO	M	DVM, former Chief Veterinary Officer and head of veterinary Services; Delegated with OIE	International	Animal epidemiology	Veterinary public health, welfare of production animals, prevention and control of diseases, epidemiology	EP, EC, PC, PH, SO
P. Léonard	Univ	M	MD, Master in Acute Medicine, Master in Internal medicine, Master in tropical medicine, University Professor (Department of Infectious Diseases and Tropical Diseases, Liege University Hospital)	Belgium	Internal tropical medicine	Infectious diseases, immunodeficiency, tropical diseases, emerging diseases, internal medicine	PH
A. Linden	Univ	F	DVM, PhD, University Professor (Department of Infectious and Parasitic Diseases, Unit of Wildlife Health and Pathologies); Walloon Wildlife Health Monitoring Surveillance Network	Belgium	Wildlife	Wildlife, mycobacteria, bluetongue, bacteriology, pathology	EP
M. Lomba	ARSIA	M	DVM, Veterinary Diagnostic Laboratory	Belgium	Animal epidemiology	Diagnosis, epidemiology, cattle, communication	EC, SO
B. Losson	Univ	M	DVM, PhD, University Professor (Department of Infectious and Parasitic Diseases, Unit of Parasitology and Parasitic Diseases)	Belgium	Parasitology	Parasitology, parasitic zoonoses, vectors, biologic control, ectoparasites	EP
J. Mainil	Univ	M	DVM, PhD, University Professor (Department of Infectious and Parasitic diseases, Unit Bacteriology and Bacteriologic Diseases)	Belgium	Bacteriology	Bacteriology, pathogeny, genetics (prokaryotes), molecular epidemiology, plasmidology	EP
D. Marlier	Univ	M	DVM, PhD, Dipl, ECZM (small mammals), University Professor (Clinical Department of Small Animals and Equids, Unit of Birds, Lagomorphs and Rodents); University Vet Clinics	Belgium	Avian and lagomorphs medicine	Aviculture, rabbit farming, birds, rabbits, rodents	EP
Y. Milleman	Univ	M	DVM, Lecturer (Head of Department of Animal Productions and Public Health, Unit of Cattle and Poultry Diseases); Unit of Food Microbiology - Safety and Quality	France	Pathology of ruminants	Cattle, *Salmonella* spp., pathology of ruminants	EP, PC, EC
B. Moinet	Wallonia	M	DVM, Cabinet of Ministry of Agriculture	Belgium	Agriculture economy	Agriculture politics, agriculture economy, ministry of agriculture	EC, SO
J.-L. Moyen	Dep, Lab	M	DVM, Head of Dordogne Departmental Laboratory	France	Laboratory diagnosis	Tuberculosis, interferon, immunoserology, ruminants, PCR	PC
P. Mullier	CVO	M	DVM, Belgian Federal Agency for the Safety of the Food Chain, Director or French-speaking and German-speaking communities	Belgium	Control	Veterinarian, public function, sanitary policy, epidemiologic surveillance, epidemiologic vigilance	PC
B. Nicks	Univ	M	DVM, PhD, University Professor (Department of Animal Productions, Unit of Veterinary Ecology and Ethology)	Belgium	Animal welfare and ethics	Animal husbandry, environment, animal welfare, animal health	SO
L. Plee	FAO	M	DVM, Epidemiologist, Animal Health Service (AGAH) and ECTAD Technical Staff, situation officer at the CMC-AH, FAO	International	Animal epidemiology	Epidemiology, zoonoses, risk assessment, veterinary legislation, subacute encephalopathies	PC, SO
A. Raskin	CVO	M	DVM, Belgian Federal Agency for the Safety of the Food Chain	Belgium	Control	Classical swine fever, stamping out diseases, identification, brucellosis, database	PC
J.-M. Robijns	CVO	M	DVM, Belgian Federal Agency for the Safety of the Food Chain	Belgium	Control	Database management, animal identification and recording, animal products and by-products traceability, control of animal diseases, support programs	PC
B. Soumaré	Univ	M	DVM, MSc, PhD; Regional Influenza Advisor, USAID West Africa Office	Belgium	Animal epidemiology	Zoonoses, pandemic threats, epidemiology, risk analysis, socioeconomic analysis	PC
J. Tafforeau	ISP	M	MD, Scientific Institute of Public Health, Head of Unit Public Health and Surveillance	Belgium	Human epidemiology	Epidemiology, chronic diseases, health determinants, investigations, health priorities	PH
E. Thiry	Univ	M	DVM, PhD, University Professor (Department of Infectious and Parasitic Diseases, Unit of Virology and Viral Diseases)	Belgium	Virology	Virus, animal, emerging diseases, genetics	EP
M. Vandenheede	Univ	M	DVM, PhD, Lecturer (Department of Animal Productions, Unit of Veterinary Ecology and Ethology)	Belgium	Ethology and animal welfare	Domestic animals, behavior, welfare, ethology	SO
L. Vanholme	CVO	M	DVM, Belgian Federal Agency for the Safety of the Food Chain	Belgium	Control	Zoonoses, reporting, animal health monitoring, animal health eradication, emerging disease	PC
P. Vannier	ANSES	M	DVM, ANSES, Head of Animal Health and Welfare	France	Animal epidemiology	Animal health, virology, epidemiology, risk analysis, vaccinology	EP, EC, PC, PH, SO
S. Zientara	INRA	M	DVM, Master in Molecular Virology, Master in Epidemiology, PhD, Central Laboratory of Veterinary Research, Maisons-Alfort Topic (Equine Viral Diseases); Head of Virology and of the National Reference Laboratory for Foot-and Mouth Disease, Bluetongue, West Nile and African Horse Sickness	France	Virology	Foot-and-mouth disease, bluetongue, West Nile fever, equine viral diseases	EP

*ID, identification; univ, University; DVM, Doctor of Veterinary Medicine; PhD, Doctor of Philosophy; EP, epidemiology; min, Ministry; EC, economy/trade; lic, license; SO, society; MD, Medical Doctor; PH, public health; Lab, Laboratory; EuroC, European Commission; FAO, Food and Agriculture Organization; PC, prevention/control; Dipl, diplomate; EVPC, European Parasitology Veterinary College; CVO, chief veterinary officer; RESPE, Réeseau d’Epidemio-Surveillance en Pathologie Equine; ANSES, French Agency for Food, Environmental and Occupational Health and Safety; OIE, World Organization for Animal Health; ARSIA, Regional Association of Animal Health and Identification; ECZM, European College of Zoological Medicine; Dep, Department; AGAH, Animal Production and Health Division of FAO; ECTAD, European Centre for Transboundary Animal Diseases; CMC-AH, Crisis Management Centre for Animal Health of FAO; USAID, United States Agency for International Development; ISP, Institute of Public Health; INRA, Institut National de la Recherche Agronomique. †International organizations.

Opinions of experts on proposed criteria were taken into account to adapt the list of criteria. For example, in the EP category, 3 rates were proposed to experts: morbidity (illness), mortality, and case-fatality. The experts suggested deleting mortality rate because case-fatality rate better reflects the gravity of the disease. Some modifications (clarifications) were made to definitions of each criterion and its coefficients. The final database of criteria, their coefficients, and definitions used for prioritization are shown in Table 1. The relative weight of each category of criteria was 20 points (mean and median) for EP, 19 (mean) and 18 (median) for PC, 23 (mean) and 28 (median) for EC, 25 (mean and median) for PH, and 14 (mean and 15 (median) for SO.

**Figure 1:**
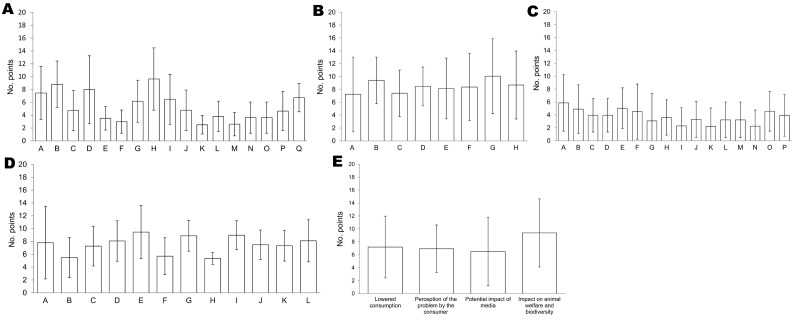
Weighting (mean no. points) of criteria for diseases of food-producing animals and zoonoses for 5 aspects of a pathogen proposed by experts, Europe. A) Epidemiology by 18 experts. A, illness rate; B, case-fatality rate; C, specificity of agents; D, mode of transmission; E, incubation period; F, clinical course; G, environmental persistence; H, epizootic potential; I, evolutive potential; J, cattle; K, small ruminants; L, swine; M, equines; N, poultry; O, lagomorphs; P, wildlife; Q, vector(s) or reservoir(s) in the European Union. B) Prevention/control by 16 experts. A, control of reservoir(s)/vector(s); B, vaccination; C, treatment; D, availability/quality of diagnostic tools; E, knowledge of pathogen; F, effectiveness of control; G, effectiveness of prevention; H, surveillance of pathogen. C) Economy/trade by 14 experts. A, loss of productivity; B, costs of mandatory slaughtering; C, costs of treatment and disinfection; D, costs of vaccination; E, limitation of importation-exportation; F, disturbance of supply/demand; G, impact on related sectors; H, impact on cattle industry; I, impact on small ruminants industry; J, impact on swine industry; K, impact on equine industry; L, impact on poultry industry; M, impact on rabbit industry; N, impact on wildlife industry; O, zoonotic impact (cost of illness); P, zoonotic impact (cost of prevention). D) Public health by 10 experts. A, zoonotic/common agent; B, classification of zoonoses; C, disease knowledge in humans; D, illness rate; E, case-fatality rate; F, contamination route; G, after effects; H, existing control plan; I, epidemic potential; J, vaccination; K, treatment; L, availability and quality of diagnostic tools. E) Society by 13 experts. Error bars indicate ± SD.

Public health was considered the major category of criteria in terms of prioritization. Weighting of criteria as proposed by experts is shown in Figure 1. Epizootic potential and case-fatality rate (%) were regarded as the 2 major epidemiologic indicators (Figure 1, panel A). Effectiveness of prevention and vaccination were weighted as the 2 major PC criteria (Figure 1, panel B). Loss of productivity and limitation of importation and exportation were the 2 major EC criteria (Figure 1, panel C). Case-fatality rate and epidemic potential were weighted as the 2 major PH criteria (Figure 1, panel D). Effect on animal welfare and biodiversity and lower consumption were the 2 major SO indicators (Figure 1, panel E). Nevertheless, within each category, differences between criteria were scarce. Conversely, the range in weights of each criterion was large, as shown by high SDs, which indicated variability among opinions of experts. To take variability into account, we used a probabilistic method to estimate the overall weighted score per disease.

### Ranking of Diseases

**Figure 2:**
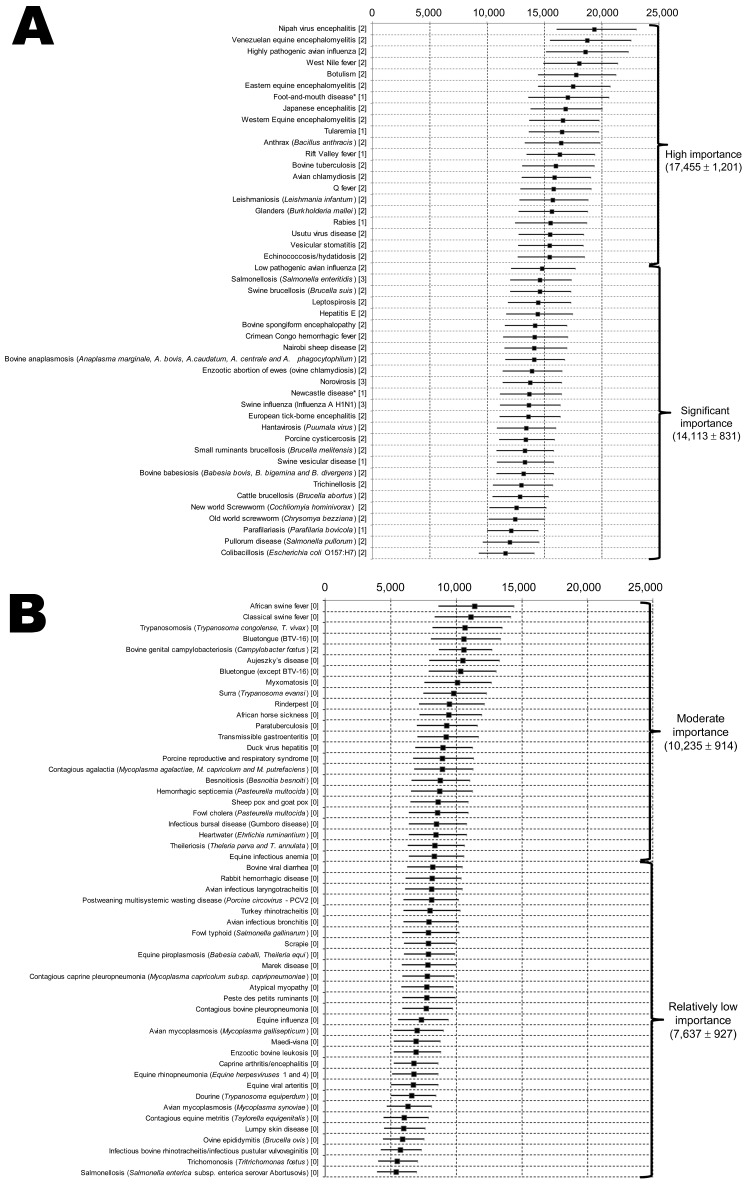
Classification and regression tree analysis showing grouping of diseases of food-producing animals and zoonoses into 4 subgroups by using overall weighted scores per disease as input, Europe. A) High importance and significant importance. B) Moderate importance and relatively low importance. Numbers at the top indicate overall weighting scores of pathogens. Squares indicate means, and error bars indicate 95% CIs. Causative agents are shown in parentheses on the left. Values on the left in brackets are zoonotic aspects codes: 2, rare; 1, accidental; 3, frequent; 0, nonzoonotic/common. Values on the right are mean ± SD weighting scores. *Foot-and-mouth disease and Newcastle disease were considered zoonotic in this study.

Final ranking of diseases according to their overall weighted scores and use of a probabilistic method is shown in Figure 2. Few differences were observed between deterministic (mean of each weight) and probabilistic methods (function of weights) (Pearson correlation coefficient 0.999; p<0.0001). This finding is probably associated with a few problems in subjective interpretation or dilution of individual discordances among the large number of experts.

FMD was considered as a zoonotic disease. However, FMD could be included in the nonzoonotic category. In such an instance, it would be the highest ranked nonzoonotic disease. Newcastle disease was included in the zoonotic/common category, which could be questioned because of its limited effect on PH. The top 5 ranked diseases were all zoonotic/common: Nipah virus encephalitis, Venezuelan equine encephalitis, influenza caused by highly pathogenic avian influenza virus, West Nile fever, and botulism.

### Classification of Diseases by Using CART Analysis

Regression trees enabled identification of 4 groups of diseases. These diseases are shown in Figure 2.

## Discussion

Prioritization of diseases has acquired major interest within the past few years, especially from a prevention point of view and in the sector of public health. Such a method is needed within the context of emerging diseases because it is not known how severe socioeconomic consequences of outbreaks will be. Our study included not only zoonoses, such as those reported by Cardoen et al. (*11*) and Havelaar et al. (*12*), but also transmissible diseases common to humans and animals and reportable animal diseases. The prioritization method was developed by an independent group to avoid any bias that could result from the influence of stakeholders as reported (*25**,**26*).

Several groups have proposed a prioritization method that considers different categories of criteria. Previous studies focused on a specific aspect of infectious diseases, such as the multicriteria analysis designed by Mourits et al., to support discussions on control measures (*27*). Conversely, the method developed by the French Agency for Food, Environmental and Occupational Health and Safety included major aspects of a disease (*28*) and also considered 2 rankings, 1 for animal health and 1 for human health. Nevertheless, our study provides a unique ranking that included both types of diseases.

The current method included more diseases compared with previous priority methods, such as those reported by DEFRA) (n = 25) or the European Commission (n = 46) (*13*). Krause et al. applied a Delphi method for collecting opinions, but only prioritized 85 zoonotic or common diseases (*9**,**10*). In our study, a Delphi method was initially planned but for time, rationale, and economic reasons, criteria were defined and established before being proposed to experts. Because our method includes qualitative and semiquantitative criteria, it is not completely numerically based, in contrast to the multicriteria analysis developed by Kurowicka et al. (*5*). Their method is applicable only for quantitative criteria and not always to all diseases in all contexts. Furthermore, they used a limited number of attributes for pathogens. Even if one relies on a quantitative method, which is less arbitrary than a semiquantitative method, the model developed by Havelaar et al. (*12*) is based on criteria reflecting the epidemiology and societal effect of zoonoses but does not include risk perception by the general public or diseases targeting animal species.

Our new method requires multidisciplinarity, which involves animal and human epidemiologists; chief veterinary officers; experts in agricultural economics, animal welfare, and biodiversity; and experts on societal aspects of diseases. Other prioritization methods often restricted their panel of experts to epidemiologists and infectious disease specialists (*9**,**10*).

The decision to start with the disease and not the animal species is in contrast to the method developed by Heffernan, who suggested that errors might be amplified throughout the weighting method (*29*). Nevertheless, by starting with the disease, the role of species is balanced by the EC category because the effect of different industries is taken into account. If an industry is not well developed in a specific area, the effect will be minimized. When the prioritization method is started with the species and its particular effect in the area/country, it makes the model applicable only in this specific area. However, if one starts with the disease and takes into account the economic role of the species in another category of criteria, the model can be applied anywhere.

Some methods applied a weighting system to criteria (DEFRA) (*9**,**10**,**30*) because it is not appropriate to consider all criteria on the same scale. For example, the human case-fatality rate should not be placed on the same scale as classification of zoonoses. Even if individual experts differed in their views on the relative role of various criteria and indicators, veterinary epidemiologists and experts in public health reached the conclusion that the epizootic/epidemic potential and case-fatality rate were the 2 major criteria in their respective category of expertise.

When one considers overall ranking of diseases, all top 20 diseases are zoonotic/common, which is expected because their global score involves the whole public health aspect. CART analysis also illustrates the correlation between PH and SO, which is not surprising because consumer behavior might be influenced when a zoonotic/common agent is involved (*31*). Nevertheless, CARTs might lead to slightly biased results in relation to variable selection: identification of distinct subgroups does not enable estimation of net effects of independent variables because subdivision of data into 2 groups is based on only 1 value of only 1 explanatory variable (*32*). In addition, bootstrap or jackknifing analysis would have been alternative ways to estimate the uncertainty.

The analysis can be applied only to the 100 diseases included in the model. Nevertheless, its predictive value is useful. The model we developed could be presented as generic and should not be confined to the 100 diseases included in the current application or to exotic diseases as with the method developed by French Agency for Food, Environmental and Occupational Health and Safety (*28*). At the beginning of the 21st century, a scientific team in the United Kingdom established a list of 1,415 pathogens that possibly affected humans (*33*). If added to the pathogens involved in animal diseases, all of these pathogens could also be added to the prioritization method, with a preliminary categorization step. As specified in the work performed under the aegis of the European Council, the prioritization exercise should be performed regularly as the epidemiologic situation of diseases constantly evolves: biotechnological improvements are constantly achieved in terms of vaccination, treatment, and diagnostic tests (*30*). In addition, elaboration of each criterion relied on evidence-based medicine through consultation with >1,800 scientific references (*34*; S. Vandeputte et al., unpub. data). The critical point of our method relies on the possible lack of independence between some criteria. Several of these criteria might be substantially dependent on each other. Although coefficients for ranges of illness and case-fatality rates were arbitrarily fixed, which may results in a loss of precision, they were accepted by experts. A Delphi method would have been more appropriate for reaching a consensus on the criteria to be used.

In conclusion, the current method is a generic tool applicable on different geographic scales in a variety of contexts because it is not restricted to well-defined field of actions. The standardization of criteria ensures transparency and reproducibility of the model in other context and for other diseases. It enables adaptations (vaccination becoming available, increased knowledge of a pathogen, viral mutations or genetic reassortments increasing host specificity). In the same view, the model could be applied to diseases affecting domestic (dogs, cats) pets or exotic pets (reptiles). Conversely, it could also be used with enzootic conditions to better retarget the surveillance system and readapt control measures worldwide.

## References

[1] Teufel P, Hammer P. Which zoonosis is it? [in Dutch]. Dtsch Tierarztl Wochenschr. 1999;106:311–8. 10488634

[2] Acha PN, Szyfres B, eds. Preface of the first English edition. In: Zoonoses and communicable diseases common to man and animals. Vol. 1: Bacteriosis and mycoses [in French]. 3rd ed. Paris: World Organisation for Animal Health; 2005. p. ix.

[3] European Commission. Health and Consumer Protection – Directorate-General, 2007. A new animal health strategy for the European Union (2007–2013) where “prevention is better than cure.” Communication from the Commission to the Council, the European Parliament, the European Economic and Social Committee, and the Committee of the Regions (COM 539 final) [cited 2011 Jan 6]. http://ec.europa.eu/food/animal/diseases/strategy/docs/animal_health_strategy_en.pdf.

[4] Organisation for Animal Health. Phylum study: listing and categorisation of priority animal diseases, including those transmissible to humans—Mission Report 2010 [cited 2011 May 16]. http://www.oie.int/fileadmin/Home/eng/Support_to_OIE_Members/docs/ppt/OIE_study_priori-catego_mission_report.pdf.

[5] Kurowicka D, Bucura C, Cooke R, Havelaar A. Probabilistic inversion in priority setting of emerging zoonoses. Risk Anal. 2010;30:715–23. 10.1111/j.1539-6924.2010.01378.x 20345579

[6] Losson B, Saegerman C. First isolation of *Parafilaria bovicola* from clinically affected cattle in Belgium. Vet Rec. 2009;164:623–6. 10.1136/vr.164.20.623 19448255

[7] European Centre for Disease Prevention and Control. Annual epidemiological report on communicable diseases in Europe 2010. Stockholm: The Center [cited 2011 May 16]. http://www.ecdc.europa.eu/en/publications/Publications/1011_SUR_Annual_Epidemiological_Report_on_Communicable_Diseases_in_Europe.pdf 22114980

[8] Berríos EP. Foot-and-mouth disease in human beings. A human case in Chile [in Spanish]. Rev Chilena Infectol. 2007;24:160–3. 17453077 10.4067/s0716-10182007000200013

[9] Krause G; Working Group on Prioritization at the Robert Koch Institute. How can infectious diseases be prioritized in public health? A standardized prioritization scheme for discussion. EMBO Rep. 2008;9:S22–7. 10.1038/embor.2008.76 18578019 PMC3327548

[10] Krause G; Working Group on Prioritization at the Robert Koch Institute. Prioritization of infectious diseases in public health—call for comments. Euro Surveill. 2008;13:pii:18996. 18831949 10.2807/ese.13.40.18996-en

[11] Cardoen S, Van Huffel X, Berkvens D, Quoilin S, Ducoffre G, Saegerman C, Evidence-based semiquantitative methodology for prioritization of foodborne zoonoses. Foodborne Pathog Dis. 2009;6:1083–96. 10.1089/fpd.2009.0291 19715429

[12] Havelaar AH, van Rosse F, Bucura C, Toetenel MA, Haagsma JA, Kurowicka D. Prioritizing emerging zoonoses in the Netherlands. PLoS ONE. 2010;5:e13965. 10.1371/journal.pone.0013965 21085625 PMC2981521

[13] Discontools project. Development of the most effective tools to control infectious animal diseases. 1Methods to the prioritization of diseases: a worldwide review of existing methodologies for health priority settings 2010 [cited 2011 Jan 5]. http://www.discontools.eu/documents/1232_Review%20of%20existing%20methodologies%20for%20priority%20settings%20-%20Draft2.pdf

[14] Sackett DL, Rosenberg WM, Gray MJ, Haynes RB, Richardson WS. Evidence-based medicine: what it is and what it isn’t. BMJ. 1996;312:71–2. 10.1136/bmj.312.7023.71 8555924 PMC2349778

[15] Kahn RE, Clouser DF, Richt JA. Emerging infections: a tribute to the one medicine, one concept. Zoonoses Public Health. 2009;56:407–28. 10.1111/j.1863-2378.2009.01255.x 19486315

[16] Radostitis O, Gay GC, Hinchcliff KW, Constable PD, eds. Veterinary medicine. A textbook of the diseases of cattle, sheep, goats, pigs and horses. 10th ed. London: Saunders-Elsevier; 2007.

[17] Kahn CM, Line S, eds. Merck Veterinary Manual. 10th ed. Whitehouse Station (NJ): Merck & Co., Inc.; 2010.

[18] Gore SM. Biostatistics and the Medical Research Council. Medical Research Council News. 1987;35:19–20.

[19] Department for Communities and Local Government, ed. Multi-criteria analysis: a manual. London: The Department; 2009 [cited 2011 Jan 7]. http://eprints.lse.ac.uk/12761/1/Multi-criteria_Analysis.pdf

[20] Breiman L, Friedman JH, Olshen RA, Stone CJ, eds. Classification and regression trees. Pacific Grove (CA): Chapman and Hall Publisher; 1984.

[21] VanEngelsdorp D, Speybroeck N, Evans JD, Nguyen BK, Mullin C, Frazier M, Weighing risk factors associated with bee colony collapse disorder by classification and regression tree analysis. J Econ Entomol. 2010;103:1517–23. 10.1603/EC09429 21061948

[22] Saegerman C, Speybroeck N, Vanopdenbosch E, Wilesmith JW, Berkvens D. Decision support tools for clinical diagnosis of disease in cows with suspected bovine spongiform encephalopathy. J Clin Microbiol. 2004;42:172–8. 10.1128/JCM.42.1.172-178.2004 14715749 PMC321688

[23] Speybroeck N, Berkvens D, Mfoukou-Ntsakala A, Aerts M, Hens N, Van Huylenbroeck G, Classification trees versus multinomial models in the analysis of urban farming systems in central Africa. Agric Syst. 2004;80:133–49. 10.1016/j.agsy.2003.06.006

[24] Steinberg D, Golowya M. CART—classification and regression trees. User’s guide 2007, San Diego: Salford Systems; 2007.

[25] Scoones I, Wolmer W. Livestock, disease, trade and markets: policy choices for the livestock sector in Africa. Institute of Development Studies Working Paper 269. Brighton (UK): Institute of Development Studies, Brighton; 2006 [cited 2011 Jan 7] ftp://ftp.fao.org/docrep/nonfao/LEAD/af853e/af853e00.pdf

[26] Perry B, Sones K. Science for development: poverty reduction through animal health. Science. 2007;315:333–4. 10.1126/science.1138614 17234933

[27] Mourits MC, van Asseldonk MA, Huirne RB. Multi-criteria decision making to evaluate control strategies of contagious animal diseases. Prev Vet Med. 2010;96:201–10. 10.1016/j.prevetmed.2010.06.010 20633939

[28] French Agency for Food. Environmental and Occupational Health and Safety (ANSES). Methodology of prioritization of animal diseases; application to the example of pathogens exotic to metropolitan France [in French]. Report 2008-SA-0390, 2010 [cited 2011 Jan 6]. http://www.afssa.fr/Documents/SANT2008sa0390.pdf

[29] Heffernan C. Panzootics and the poor: devising a global livestock disease prioritization framework for poverty alleviation. Rev Sci Tech. 2009;28:897–907. 20462148

[30] Council of the European Union. EU animal health strategy: non-paper on prioritization of animal-related threats and biosecurity. Presented at: Second Meeting of CVO Working Party # 1. DG B I JR:ddc – 9536/08 ADD 1; 2008 May 22; Brussels. p. 1–8.

[31] Frank C, Faber M, Askar M, Bernard H, Fruth A, Gilsdorf A, Large and ongoing outbreak of haemolytic uraemic syndrome, Germany, May 2011. Euro Surveill. 2011;16:pii:19878. 21632020

[32] Speybroeck N. Classification and regression trees. Int J Public Health. 2011 Oct 21; [Epub ahead of print]. 10.1007/s00038-011-0315-z 22015650

[33] Taylor LH, Latham SM, Woolhouse ME. Risk factors for human disease emergence. Philos Trans R Soc Lond B Biol Sci. 2001;356:983–9. 10.1098/rstb.2001.0888 11516376 PMC1088493

[34] Vandeweerd JM, Kirschvink N, Clegg P, Vandenput S, Gustin P, Saegerman C. Is evidence-based medicine so evident in veterinary research and practice? History, obstacles and perspectives. Vet J. 2012;191:28–34. 10.1016/j.tvjl.2011.04.013 21620746

